# Burden and presentation of depression among newly diagnosed individuals with TB in primary care settings in Ethiopia

**DOI:** 10.1186/s12888-017-1231-4

**Published:** 2017-02-07

**Authors:** Fentie Ambaw, Rosie Mayston, Charlotte Hanlon, Atalay Alem

**Affiliations:** 1 Bahir Dar University, School of Public Health, Bahir Dar, Ethiopia; 2 Department of Psychiatry, Addis Ababa University, College of Health Sciences, School of Medicine, Addis Ababa, Ethiopia; 3 King’s College London, Institute of Psychiatry, Psychology and Neuroscience, Centre for Global Mental Health, London, UK

**Keywords:** Depression, Tuberculosis, Primary care, Ethiopia

## Abstract

**Background:**

Understanding co-morbidity of depression and tuberculosis (TB) has been limited by challenges in measurement of depression due to overlapping symptoms, use of small hospital samples and uncontrolled analysis. This study was conducted to better understand the burden and presentation of depression, and associated factors in people with TB in primary care settings in Ethiopia.

**Methods:**

We conducted a cross-sectional survey among 657 people newly diagnosed with TB. Symptoms of depression were measured using the Patient Health Questionnaire (PHQ-9). TB symptoms and other factors were captured using standardised questionnaires. The factor structure of PHQ-9 was examined. Multivariable analysis was carried out to estimate prevalence ratios.

**Results:**

The prevalence of probable depression was 54.0%. The PHQ-9 had one factor structure (alpha = 0.81). Little interest or pleasure in doing things (73.0%) was the commonest depressive symptom. Older age (Adjusted Prevalence ratio (APR) = 1.19; 95%CI = 1.06, 1.33), female sex (APR = 1.23; 95%CI = 1.18, 1.27), night sweating (APR = 1.25; 95%CI = 1.16, 1.35), pain (APR = 1.69; 95%CI = 1.24, 2.29), being underweight (APR = 1.10; 95%CI = 1.07, 1.13), duration of illness (APR = 1.35; 95%CI = 1.22, 1.50), level of education (APR = 0.93; 95%CI = 0.90, 0.95), and social support (APR = 0.89; 95%CI = 0.85, 0.93) were independently associated with probable depression.

**Conclusions:**

Depression appears highly prevalent in people with TB and PHQ-9 seems to be a useful instrument to detect depression in the context of TB. The frequency of depressive symptoms would suggest that the occurrence of the symptoms in people with TB is in the usual manifestation of the disorder. Prospective studies are needed to understand the longitudinal relationship between TB and depression.

## Background

Depression and tuberculosis are important global public health concerns, contributing 2.5% and 2.0%, respectively to Global Burden of Disease (GBD), as measured by Disability Adjusted Life Years (DALYs) in 2010 [1]. In the same year, depression together with other mental and substance use disorders contributed 7.4% to the total DALYs; TB together with HIV/AIDS contributed 5.3% to the total DALYs [2]. Both depression and TB have substantial contributions to the burden of disease in Ethiopia. Prevalence estimates of depression range from about 5% in large sample population studies [3, 4] to 9.1% in a nationally representative sample [5]. Ethiopia is one of the 22 countries that collectively account for 81% of all cases and 80% of all deaths from TB worldwide [6]. In 2013, TB was the fourth highest contributor to DALYs in the country [7].

Evidence from cross-sectional studies in both high income [8] and low and middle income countries [9–17] indicates a very high prevalence of co-morbid depression among people with TB. The direction of causality is currently unclear and is likely to be complex. However, a large longitudinal study in Taiwan found that newly diagnosed pulmonary TB patients on treatment were at higher risk of developing depression compared to healthy controls after a mean period of 6.5 years [18]. Co-morbid depression worsens the course of medical disorders and has an adverse impact upon the quality of patient-physician relationships [19]. People with co-morbid depression are less satisfied with their primary care physicians, making more health care visits for vague symptoms but delaying visits for important medical problems and missing essential scheduled visits [20]. Little is known about the burden and impact of co-morbidity in Ethiopia.

In fact, overall, the evidence base on TB and depression is sparse. The design of published studies, many of which are small, cross-sectional and carried out among inpatients, limits interpretation and generalisability of findings. For example, hospital samples consist of severely ill patients with additional significant stress from being hospitalized; therefore, conclusions may not apply to primary care patients. All previous studies included TB patients on unspecified medication which could lead to confounding. Finally, the challenge of overlapping symptoms of somatised depression and TB has not been adequately addressed. Weight loss, loss of appetite, and fatigue are characteristic of both TB and somatic symptoms of depression [21]. The latest Diagnostic and Statistical Manual of Mental Disorders (DSM-5) states that such symptoms may not count towards a major depressive disorder diagnosis when they are “clearly and fully attributable” to a general medical condition [22]. Given that co-morbid depression has been shown to be associated with a range of adverse outcomes [20], we would argue that it is important to try to separate depressive symptoms from those of TB, in order that, ultimately, these may be detected and treated in the context of TB services.

To better understand the burden and presentation of depression in the context of TB, the objectives of this study were to: a) determine the prevalence of depression; b) examine the relative frequency of individual depressive symptoms, and d) investigate associations with socio-demographic and TB-related factors.

## Methods

### Design

We present cross-sectional baseline data from an ongoing cohort study investigating the interaction between depression and tuberculosis in primary care settings in Ethiopia. For a detailed description of methodology see the published protocol [23].

### Study setting

The study was conducted in five districts of the Southern Nations, Nationalities and Peoples’ Region (SNNPR) of Ethiopia namely, Butajira town, Mareko, Meskan, and Sodo districts of Gurage zone, and Silti district of Silte zone from December 2014 to January 2016. We had expected to find enough sample size in the above districts in the specified study period, but due to lower than expected numbers of new cases of TB in these sites, we included five additional primary care centres from Bahir Dar and Woreta towns of Amhara National Regional State in the Northwest part of the country from June 2015.

Integrated mental health services were available in all of the primary care centres included in this study. Nurses and health officers are the main clinicians in these settings that provide curative services to the community. All of these health workers in these health facilities had received brief training in the management of priority mental disorders according to the evidence-based WHO Mental Health Gap Action Programme Intervention Guide (mhGAP-IG) for mental, neurological and substance use disorders in non-specialized health care settings [24].

### Inclusion criteria

- People attending the selected health centres for TB treatment who were within one month of starting anti-TB treatment
- Aged 18 years and above

### Exclusion criteria

- People planning to move out of the study area
- Those too ill to be interviewed at baseline: as perceived by the interviewer or the prospective participant
- Those who were admitted to in-patient unit for more than 5 days in the last 1 month: as the additional stressors of being hospitalized represent a different range of risk factors for depression.
- People with MDR-TB, who constitute a different population because their treatment and outcomes are different (more toxic medications for a much longer duration and poorer prognosis) and MDR-TB is a more feared and stigmatised condition [25]. Furthermore, only one of the study health facilities had recently started a service for people with MDR-TB.
- People on re-treatment for TB, who had experienced previous treatment failures, were at high risk of MDR-TB and constituted a different risk group for depression.

### Sample size determination

The sample size was based on the primary objective of the planned longitudinal study which was to examine the effect of depression on default from anti-TB treatment [23] and was calculated using the following parameters: 80% power, 95% confidence level, 2.5% prevalence of treatment default among patients with TB and without depression, 7.5% prevalence of treatment default among people with TB and co-morbid depression, a ratio of 2:1 of non-exposed (not depressed) to exposed (depressed) participants (*n* = 639) and a contingency of 10% for possible loss to follow up. This gave a required sample size of 703 people with TB. A total of 657 participants were recruited due to scarcity of cases that met our selection criteria.

### Sampling technique

All illegible individuals were invited to participate in the study consecutively until the required sample size was reached. A total of 965 individuals visited the selected health institutions for TB care during our study period (13 months). Three hundred four of them were excluded using our exclusion criteria: under age (*n* = 145), plan of transfer out (*n* = 21), taken anti-TB medications for more than one month (*n* = 31), admitted for more than five days over the last one month (*n* = 30), confirmed to have MDR-TB (*n* = 11), on retreatment regimen (*n* = 57), and did not speak the language (*n* = 9). Of the 661 individuals illegible for the study, four individuals (0.61%) refused to participate in our study. The long duration of consecutive recruitment from multiple health facilities with almost all illegible individuals included makes our sample fairly representative of adults newly diagnosed with TB.

### Recruitment and data collection

The clinicians running TB outpatient clinics screened people for eligibility, explained the research project and invited them to participate in the study. When the person expressed interest to participate, the workers linked them to a trained nurse research assistant who gave further detailed information, sought written consent and carried out the baseline interviews. The nurse research assistants were fulltime employees responsible for data collection. Interviews were mostly conducted at the primary care health facility using a structured and pre-coded questionnaire translated into Amharic. A small number of participants (*n* = 13) were interviewed at their homes as this was their expressed preference.

The clinicians and the nurse research assistants received a five days training on: how to approach respondents and obtain consent; administration of questionnaires, and ethical principles of research. The training involved role plays and practice with people who were attending TB clinic. Video records of the role plays were used to facilitate learning. A further five days refresher training was given after six months.

### Variables and measurement

The dependent variable was probable depression measured using the Patient Health Questionnaire (PHQ-9). PHQ-9 is in widespread use in both research and clinical practice. The scale has been used in very large surveys, randomized effectiveness trials and cohort studies as well as in different settings and population groups [26]. We have used the term depression to mean probable depression for the purpose of simplicity.

The Amharic version of the scale has been formally validated in Ethiopia in two separate studies. The first validation was conducted in a large sample of medical out-patients in a tertiary referral hospital in Addis Ababa [27]. In that study, PHQ-9 was found to be unidimensional (one factor structure) and, at an optimal cut-off point for major depressive disorder (MDD) of 10 and above, sensitivity was 86% and specificity 67% [27]. The second PHQ-9 validation was conducted in attendees of primary care out-patient clinics in a predominantly rural area [28]. In this study, PHQ-9 had a sensitivity of 0.83 and specificity of 0.75 for detection of major depressive disorder (MDD) at a cut-off value of five or more [28]. In the current study, we opted to use the more conservative cut-off point of ten and above to define probable depression because of concerns about overlap of symptoms between tuberculosis and the somatic symptoms of depression included in PHQ-9 (loss of appetite, weight loss and fatigue) [21].

### Independent variables

#### Socio-demographic variables

Age, sex, marital status, level of education, religion, household income, occupation, and place of residence (urban versus rural) were socio-demographic independent variables used in the analysis. Household income was measured by asking the participants to estimate the monthly total income of their household. When the participant was a farmer, we changed the estimates of annual income in kind to cash using the local market price. We converted the monthly income into annual income.

#### Symptoms of tuberculosis

Fever, night sweating, anorexia, fatigue, cough, and pain were each scored from zero (absence of symptom) to ten (worst level of symptom severity) as perceived by the respondent for that week. The responses were then categorised as follows: no symptom (0), mild (1 to 4), moderate (5 to 6), and severe (7 to 10), using a visual analogue scale to indicate intensity, as recommended by Jones et al. [29]. The duration of TB symptoms was captured by asking the respondents “how long did your TB symptoms last before your illness was diagnosed as TB?”

#### Duration of anti-TB treatment

It was calculated from the date of starting the medications which was found from the standard TB register and the date of interview.

#### Body Mass Index (BMI)

Weight and height were measured using standard procedures. BMI was calculated as weight (kilograms)/height (metres^2^). BMI values < 18.5 kg/m^2^ were defined as underweight [30].

#### Substance use

Alcohol, tobacco and khat use were measured using the WHO Alcohol, Smoking and Substance Involvement Screening Test (ASSIST) (version 3.1) [31]. ASSIST was designed for use across different cultural settings. The instrument’s psychometric properties have been tested using data from multiple countries, including low, middle, and high income countries and shown to be valid, reliable and easy to administer across settings [32]. The ASSIST risk score ranges from 0 to 31 for tobacco and 0–39 for alcohol and khat. The risk score of the respondents obtained for alcohol is categorised into ‘low’ (0 to 10), ‘moderate’ (11 to 26) or ‘high’ risk (above 26), and for khat low (0 to 3), moderate (4 to 26) and high (above 26) [31].

#### Co-morbid illness

Data on the presence of chronic illnesses other than TB were obtained by asking the question “Have you ever been told by health professionals to have cardiac illness, hypertension, diabetes mellitus, depression, or mental illnesses other than depression?” HIV status was recorded from the TB register after informed consent.

#### Perceived social support

Perceived social support was measured using the three-item Oslo Scale of Perceived Social Support with scores ranging from 3 to 14 [33]. The scale was previously reported to work well in Ethiopia in TB patients [15]. The Z-score of the scale total was cut into three equal parts and then operationalized as poor, moderate and strong perceived social support.

### Data management

We have followed Strengthening the Reporting of Observational Studies in Epidemiology (STROBE) guidelines for cross-sectional studies [34]. All instruments were piloted in 68 people with TB who were not included in the main study where clarity, acceptability, and feasibility of the baseline questionnaire were checked. The principal investigator made regular visits to the field to monitor data collection. The health institutions were visited at least once every two weeks to make sure that the data collection was going as planned, and supervision meetings were held with data collectors and supervisors at least every month. The collected data were checked for completeness and consistency by the supervisors in the field. Data were entered by the principal investigator within a week using IBM SPSS 20.0 (IBM Corp. Released 2011. IBM SPSS Statistics for Windows, Version 20.0. Armonk, NY: IBM Corp.). Accuracy of data entry was checked by running frequency analysis and making range checks every time data were entered. Errors of data entry were corrected by cross-checking with the filled questionnaires. Dates for second and third round assessments for each participant were calculated by the principal investigator using SPSS and passed on to supervisors and data collectors so that they were able to arrange data collection.

### Data analysis

Analysis was done using STATA version 13.1 (StataCorp. URL:http://www.stata.com). We have tried to determine whether the somatic symptoms of depression were measuring the same underlying construct as the cognitive and emotion symptoms. In preparation for factor analysis, we checked PHQ-9 data for presence of adequate correlations among items and adequacy of sample. We then carried out exploratory factor analysis with maximum likelihood extraction and oblimin rotation. Three criteria were used to determine the number of factors extracted: eigen value above one, scree plot and parallel analysis. Internal consistency was checked using Cronbach’s alpha and mean inter-item correlation coefficient.

The prevalence of probable depression among TB patients at baseline was determined by computing the proportion of patients scoring 10 or more on the PHQ-9 scale. As recommended for analyzing prevalence ratios in cross-sectional binary outcome studies where the outcome is common [35–37], the factors associated with depression at baseline were examined using both bivariate and multivariable Poisson regression with robust variance estimator. Because the study sites were located in two regions of the country, adjustment was made to the standard errors during analysis. The inclusion of independent variables in the multivariable analysis was based on: a) its theoretical importance and b) adequacy of the number of participants in cells for each category. In order to estimate the magnitude of variance contributed by TB symptoms above and beyond socio-demographic factors, hierarchical multiple regression was carried out by using the PHQ-9 scores as a continuous dependent variable. In this analysis, the physical symptoms of TB (cough, night sweating, BMI, duration of TB symptoms, duration of anti-TB treatment, and pain) were modelled in the second block after modelling for socio-demographic variables in the first block as recommended by Tabachnic and Fidell [38]. Although loss of appetite and fatigue are symptoms of TB, they were not included in the multivariable model because the PHQ-9 has these same items. The necessary assumptions were checked before running regression analyses.

The frequency of depressive symptoms that occurred everyday for the whole two weeks was computed and ranked.

## Results

### Socio-demographic characteristics of the participants

This study included 657 people newly diagnosed with TB, aged 18 to 85 years (median = 30; interquartile range = 16). Three hundred five (46.4%) were under 30, more than half (54.2%) were male, 227 (34.6%) were non-literate, 365 (55.6%) were married, 409 (62.3%) were Orthodox Christians, 310 (47.2%) were Amhara by ethnicity, 176 (26.8%) were farmers, 370 (56.3%) were urban residents, and 557 (84.5%) were earning less than one USD per day (Table 1).

**Table 1:** Socio-demographic characteristics of the study participants

Characteristics	Number (%)
Sex	
Male	356 (54.2)
Female	301 (45.8)
Age	
18–29 years	305 (46.4)
30–39 years	161 (24.5)
40–49 years	75 (11.4)
50–59 years	52 (7.9)
60–69 years	35 (5.3)
70–79 years	17 (2.6)
80–89 years	12 (1.8)
Level of education	
Unable to read and write	227 (34.6)
Able to read & write only	71 (10.8)
Grade 1–4	74 (11.3)
Grade 5–8	118 (18.0)
Grade 9–12	106 (16.1)
College graduate	61 (9.3)
Marital status	
Single	214 (32.6)
Married	365 (55.6)
Divorced	47 (7.2)
Widow	31 (4.7)
Residence	
Urban	370 (56.3)
Rural	287 (43.7)
Occupation	
Government employee	62 (9.4)
Unemployed	37 (5.6)
Self-employed	134 (20.4)
Farmer	176 (26.8)
Student	40 (6.1)
Housewife	111 (16.9)
Daily labourer	44 (6.7)
Others	53 (8.1)
Religion	
Orthodox	409 (62.3)
Islam	222 (33.8)
Protestant	23 (3.5)
Catholic	2 (0.3)
Other	1 (0 .2)
Ethnicity	
Amhara	310 (47.2)
Gurage	194 (29.5)
Mareko	69 (10.5)
Silte	67 (10.2)
Oromo	11 (1.7)
Other	6 (0.9)
Income	
< 1USD/person/day	557 (84.5)
≥ 1USD/person/day	100 (15.2)
Annual household income in Birr	13300 ± 14000

### Illness characteristics of the participants

Participants reported that they had been ill with symptoms of TB for 0.3 to 520.0 weeks (median = 12.0 weeks). At the time of this study, 422 (64.2%) had never taken anti-TB medication (median duration of anti-TB medication = 0; range = 0 to 27 days). In addition to TB, 75 (11.4%) had HIV, and 11 participants reported having other co-morbid chronic illnesses: hypertension (*n* = 2), cardiac illness (*n* = 3), diabetes mellitus (*n* = 5), and mental illness other than depression (*n* = 1). No participant had been diagnosed with depression prior to the study (Table 2).

**Table 2:** Illness related characteristics of the respondents

Characteristics	Number (%)
Duration of TB symptoms)	
< 2 weeks	92 (14.0)
2–12 weeks	289 (44.0)
13–52 weeks	214 (32.6)
> 52 weeks	62 (9.4)
Duration of anti-TB medication before interview)	
No medication	422 (64.2)
1–3 days	130 (19.8)
> 3 days	105 (16.0)
HIV status	
Positive	75 (11.4)
Negative	502 (76.4)
Unknown	80 (12.2)
Level of alcohol risk	
Low	568 (86.5)
Moderate	77 (11.7)
High	12 (1.8)
Level of tobacco risk	
Low	624 (95)
Moderate	29 (4.4)
High	4 (0.6)
Level of khat risk	
Low	553 (84.2)
Moderate	93 (14.2)
High	11 (1.7)

### Prevalence of depression and burden of depressive symptoms

The prevalence of probable depression in people with TB using a cut-off value of ten and above was 54.0% (95%CI = 50.2, 57.7%). The prevalence was slightly higher in women (58.1%; 95%CI = 52.5, 63.8%) compared to men (50.6%; 95%CI = 45.5, 55.6%). Little interest or pleasure in doing things (73.0%), fatigue (64.5%), appetite change (51.3%), sleep disturbance (38.9%), depressed mood (36.9%), psychomotor disturbance (27.9%), feeling bad or failure (23.4%), difficulty to concentrate (21.7%), and suicidality (8.2%) were the rank of frequency of symptoms in participants with probable depression. In participants who did not have probable depression the rank of frequency of symptoms was loss of interest (20.2%), fatigue (11.6%), appetite change (9.9%), sleep disturbance (4.6%), depressed mood (4.0%), feeling bad or failure (2.0%), psychomotor disturbance (1.0%), and difficulty to concentrate (0.3%); none of them had suicide ideation. The cognitive symptoms were observed almost exclusively in people with probable depression.

### The characteristics of PHQ-9 in TB patients

PHQ-9 had a clear single structure explaining 35.0% of the variance on the basis of the scree plot, eigen values and parallel analysis. The scale had an alpha value of 0.81, and a mean inter-item correlation coefficient of 0.33. The loadings of the items ranged from 0.50 for appetite change to 0.73 for psychomotor disturbance.

### Socio-demographic variables associated with depression

Analysis was conducted with data from 644 participants due to missing measures of BMI for 13 participants. In the bivariate analyses, female sex, older age, lower educational status, rural residence, lower household income, poor perceived social support, and marital experience (married, divorced or widowed compared to single) were associated with increased prevalence of depression (*p* < 0.01). In the multivariable model adjusted for socio-demographic variables, women had 23.0% increased risk of depression compared to men (Adjusted Prevalence Ratio (APR) =1.23; 95%CI = 1.18, 1.27). For every 14 years increase in age, the risk of having depression increased by 19.0% (95%CI = 1.06, 1.33). Attaining secondary education or higher (APR = 0.93; 95%CI = 0.90, 0.95) and strong perceived social support (APR = 0.89; 95%CI = 0.85, 0.93) were found to be negatively associated with depression (Table 3).

**Table 3:** Factors associated with depression in people with newly diagnosed TB

Characteristics	Depression status		Crude Prevalence Ratio (CPR) (95%CI)	Adjusted^a^Prevalen-ce Ratio (APR) (95%CI)
	No	Yes		
Sex				
Male	174	172	1	1
Female	124	174	1.17 (1.16–1.19)***	1.23 (1.18–1.27)***
Age (Z-score; SD = 14):	30.7 ± 11.5	37.7 ± 16.3	1.22 (1.07–1.39)**	1.19 (1.06–1.33)**
Level of education				
No formal education	105	186	1	1
Primary education	89	101	0.83 (0.82–0.85)***	1.09 (1.01–1.18)*
Secondary education or higher	104	59	0.57 (0.53–0.60)***	0.93 (0.90–0.95)***
Residence				
Urban	198	162	1	1
Rural	100	184	1.44 (1.13–1.84)**	1.22 (0.91–1.64)
Religion				
Christian	225	198	1	1
Muslim	73	148	1.43 (0.97–2.11)	1.23 (0.79–1.92)
HIV status				
Negative	235	260	1	1
Positive	31	40	1.07 (0.67–1.71)	1.33 (0.81–2.17)
Unknown	32	46	1.12 (0.95–1.32)	1.11 (0.88–1.40)
Annual household income (Z-scores; SD = 657USD):	22 USD ± 27	12.7 USD ± 18	0.78 (0.68–0.88)***	0.87 (0.74–1.02)
Marital status				
Single	124	85	1	1
Married	144	216	1.48 (1.18–1.84)**	1.06 (0.65–1.71)
Divorced or widow	30	45	1.48 (1.32–1.65)***	0.97 (0.72–1.32)
Perceived social support				
Poor	79	135	1	1
Moderate	121	112	0.76 (0.72–0.81)***	0.83 (0.79–0.88)***
Strong	98	99	0.80 (0.72–0.88)***	0.89 (0.85–0.93)***
Cough				
No cough	135	69	1	1
Mild-to-moderate cough	123	139	1.57 (0.90–2.73)	1.40 (0.92–2.12)
Severe cough	40	138	2.29 (1.07–4.90)*	1.53 (0.88–2.68)
Night sweating				
No night sweating	124	75	1	1
Mild-to-moderate night sweating	129	145	1.40 (1.11–1.77)**	1.21 (1.12–1.32)***
Severe night sweating	45	126	1.96 (1.53–2.49)**	1.25 (1.16–1.35)***
Duration of anti-TB medication				
Never taken medication	190	227	1	1
1–3 days medication	60	68	0.98 (0.76–1.26)	0.97 (0.71–1.31)
More than 3 days medication	48	51	0.95 (0.60–1.50)	1.14 (0.77–1.69)
Duration of TB symptoms:				
Less than 2 weeks	57	34	1	1
2–12weeks	121	159	1.52 (1.36–1.69)***	1.40 (1.19–1.66)***
More than 12–52 weeks	93	118	1.50 (1.15–1.94)**	1.40 (1.00–1.94)
More than 52 weeks	27	35	1.51 (1.19–1.92)**	1.35 (1.22–1.50)***
BMI				
Not underweight	154	131	1	1
Underweight	144	215	1.30 (1.02–1.66)*	1.10 (1.07–1.13)*
Pain				
No pain	37	17	1	1
Mild-to-moderate pain	195	122	1.22 (0.96–1.56)	1.09 (0.99–1.21)
Severe pain	66	207	2.41 (1.50–3.87)***	1.69 (1.24–2.29)***

*A* adjustment was made for all variables in the table; * = *p* < 0.05; ** = *p* < 0.01; *** = *p* < 0.001

### Tuberculosis symptoms and depression

In the bivariate analysis, all TB related variables except duration of anti-TB treatment were associated with depression (*p* < 0.05). In the final model where adjustment was made for socio-demographic variables and TB symptoms, night sweating (APR = 1.25; 95%CI = 1.16, 1.35), pain (APR = 1.69; 95%CI = 1.24, 2.29), underweight (APR = 1.10; 95%CI = 1.07, 1.13), and longer duration of illness with TB (APR = 1.35; 95%CI = 1.22, 1.50) were independently associated with depression (Table 3). In a hierarchical multiple linear regression analysis, TB symptoms together explained 12.4% of variance of depressive symptoms after controlling for socio-demographic variables.

## Discussion

### Prevalence of depression

The overall prevalence of probable depression was 54.0% which is higher than reported in a study that used the depression component of the hospital anxiety and depression scale which was 43.4% in Ethiopian TB patients on treatment [15]. Similar high prevalence was reported from Nigeria [9, 17], India [10] and Turkey [12] regardless of whether structured diagnostic tools [17] or screening instruments such as PHQ-9 [9] were used.

### Depressive symptoms in people with TB

The clear one factor structure and the high internal consistency of PHQ-9 support the notion that both the somatic and the cognitive-emotion symptoms of depression are measuring the same underlying construct in people with TB [39–41]. The individual item-factor correlations ranged from 0.50 to 0.73 (fair to excellent) [38]. Previous PHQ-9 validation studies from Ethiopia also support unidimensionality of the scale [27, 28]. These findings indicate that the potential overlap between TB symptoms and somatic symptoms of depression may not significantly compromise the construct validity of the tool.

Close observation of the frequency of depressive symptoms would also suggest that the occurrence of the symptoms in people with TB is in the usual manifestation of the disorder. Basically, depression is known to have multiple somatic symptoms together with psychological symptoms as important elements for diagnosis [42]. Sometimes the presenting complaints of depressed mood are insomnia [43] and fatigue [42] resulting in under diagnosis if probing is not done for accompanying depressive symptoms [22]. Because PHQ-9 does not allow probing of respondents, lower frequency of depressed mood compared to anhedonia could be expected in our study. Similarly, psychomotor disturbances are much less common in patients with depression but are indicative of greater overall severity when they exist [22]. Although the observed high frequency of somatic symptoms could be partly explained by the physical illness [44], somatic symptoms are also very likely to constitute the presenting complaint of depression [22, 42, 45] particularly when they are persistent and their onset coincides with the onset of depressed mood or anhedonia [44]. In this analysis, we considered the symptoms when they occurred throughout the two weeks prior to the survey.

### Factors associated with depression

The common factors known to be associated with depression in the general population have been found to show significant associations with depression in people with TB indicating the convergent validity of PHQ-9 in this population. Our study is in agreement with previous studies which reported that female sex [46, 47] and older age [5, 48] were positively associated with depression whereas level of education [49, 50] and perceived social support [51] were negatively associated with depression.

In agreement with studies conducted in Nigeria [17] and Greece [14], after controlling for socio-demographic factors, BMI, night sweating, pain, and the duration of these symptoms were associated with depression. In our cross-sectional study, the exact nature of the association between depression and TB symptoms is difficult to disentangle. One possible explanation is that patients may develop depression as a result of chronic TB infection, related psycho-socio-economic stressors and general factors such as weight loss [52], and the severity of medical illness [53]. Serious medical illness is a strong stressor affecting body image, self-esteem, capacity to maintain family and social relationships, and sense of loss [52, 54]. The association between depression and the severity of TB symptoms could also be because depression increases the severity of symptoms of physical illness [22]. Finally, depression and TB are likely to share risk factors, such as compromised immunity and stress [55–57]. The observed total explained variance of 12.4% by all TB symptoms together which is below the margin of moderate effect [58] may strengthen the hypothesis that TB and depression may share risk factors significantly. Although we are unable to disentangle the direction of the association between depressive and TB symptoms in our current analysis of cross-sectional data, our longitudinal data will enable us to explore the temporal relationship further in future analyses.

### Strengths and limitations

Unlike all previous studies, this study had sufficient sample size for both exploratory factor analysis and controlled regression analysis. We were able to control for the effects of anti-TB medications, as some of the participants did not take medication at all at the time of baseline assessment. In an effort to obtain a more homogenous sample, we focused on primary care patients. We assessed the relative burden of specific depressive symptoms and the usefulness of somatic symptoms of depression in the context of TB.

In addition to the cross-sectional design, we used a screening tool which has been validated in Ethiopia in outpatient populations but not validated specifically among TB patients. There is overlap between the symptoms of TB and depression. The overlap in the symptoms of TB and some items of PHQ-9 might have led to over classification of cases as depression with the resultant weakening effect on the association between depression and the independent variables. Therefore, we opted to classify our cases as high and low symptom scorers and we have used a conservative cut-off to increase its specificity. Post hoc analysis of the scale characteristics in our sample has also indicated conventional performance of the scale in our sample.

## Conclusions

We found probable depression to be highly prevalent among our sample of adult people newly diagnosed with TB in primary care settings. Our findings suggest that it may be appropriate for clinicians and researchers to include somatic symptoms of depression in their assessments of TB patients for depression provided that the affective and cognitive symptoms are present. The single factor structure of PHQ-9 results, good internal consistency and the familiar manifestations of symptomatology suggest that the PHQ-9 may be suitable for detecting depression in the context of TB. However, the predictive validity of the scale needs to be determined. The duration and severity of TB symptoms were important factors independently associated with depression after socio-demographic factors were controlled. Prospective studies are needed to understand the longitudinal relationship between TB symptoms, depressive symptoms and TB outcomes.

## Highlights

- Depression was prevalent in untreated newly diagnosed tuberculosis (TB) patients.
- The commonest symptom of co-morbid depression in TB patients was anhedonia.
- The burden of depressive symptoms in co-morbid patients was similar to the familiar manifestation of the disorder.
- Somatic symptoms show depression if affective and cognitive symptoms are there.
- TB symptoms as a group explain 12.0% of the variance in predicting depression.

## Abbreviations

ASSIST: Alcohol, Smoking, and Substance Involvement Screening Test
BMI: Body Mass Index
DALYs: Disability Adjusted Life Years
MDR-TB: Multidrug-Resistant Tuberculosis
mhGAP-IG: World Health Organization Mental Health Gap Action Programme Intervention Guide
PHQ-9: Patient Health Questionnaire, nine item version
TB: Tuberculosis
